# Proteasome inhibitors against amelanotic melanoma

**DOI:** 10.1007/s10565-017-9390-0

**Published:** 2017-03-09

**Authors:** Justyna Sidor-Kaczmarek, Mirosława Cichorek, Jan Henryk Spodnik, Sławomir Wójcik, Janusz Moryś

**Affiliations:** 1 Department of Anatomy and Neurobiology, Medical University of Gdansk, Gdansk, Poland; 2 Department of Embryology, Medical University of Gdansk, Gdansk, Poland

**Keywords:** Apoptosis, Cancer, Melanoma, Proteasome inhibitors

## Abstract

The incidence of malignant melanoma, the most aggressive skin cancer, is increasing constantly. Despite new targeted therapies, the prognosis for patients with metastatic disease remains poor. Thus, there is a need for new combinational treatments, and antineoplastic agents potentially valuable in this approach are inhibitors of the ubiquitin-proteasome system (UPS). In this work, we analyze the cytotoxicity mechanisms of proteasome inhibitors (MG-132, epoxomicin, and lactacystin) in a specific form of melanoma which does not synthesize melanin—the amelanotic melanoma (Ab cells). We found that the most cytotoxic of the compounds tested was epoxomicin. Caspase-9 activation as well as cytochrome C and AIF release from mitochondria indicated that exposure to epoxomicin induced the mitochondrial pathway of apoptosis. Epoxomicin treatment also resulted in accumulation of Bcl-2 family members—proapoptotic Noxa and antiapoptotic Mcl-1, which were postulated as the targets for bortezomib in melanoma. Inhibition of caspases by BAF revealed that cell death was partially caspase-independent. We observed no cell cycle arrest preceding the apoptosis of Ab cells, even though cdk inhibitors p21^Cip1/Waf1^ and p27^Kip1^ were up-regulated. The cell cycle was blocked only after inactivation of caspases by the pan-caspase inhibitor BAF. In summary, this is the first study exploring molecular mechanisms of cell death induced by epoxomicin in melanoma. We found that Ab cells died on the mitochondrial pathway of apoptosis and also partially by the caspase-independent way of death. Apoptosis induction was fast and efficient and was not preceded by cell cycle arrest.

## Introduction

Malignant melanoma is exceptionally difficult to treat since it metastasizes early and is resistant to conventional chemo- and radiotherapy. Although in recent years great progress has been made thanks to BRAF and MEK inhibitors and new immunotherapies (Amann et al. 2016), significant limitations remain. Despite the impressive clinical results, BRAF and MEK inhibitors can be successfully applied only in patients with respective mutations. Because of rapidly developing resistance, the median duration of responses is less than 1 year and the 5-year survival rate remains lower than 10% (Johnson et al. 2015; Tang et al. 2016). Hence, there is still a need to develop new strategies in melanoma therapy.

In this context, proteasome can be considered a promising therapeutic target. Proteasome, a multisubunit complex with protease activity, is a key element of the ubiquitin-proteasome system (UPS), which is responsible for over 80% of cellular protein degradation. Controlling the half-life of proteins engaged in apoptosis, cell cycle, transcription, DNA repair, and many other processes, UPS affects virtually all vital cell functions (Crawford et al. 2011; Cohen-Kaplan et al. 2016). Even though no clinical trials published for far on proteasome inhibitors in patients with advanced melanoma showed a significant response, the potential use of these agents as elements of combinatorial therapy in melanoma is worth investigating (Grazia et al. 2014; Obrist et al. 2015).

There are four basic groups of proteasome inhibitors. The first synthetic inhibitors discovered were peptide aldehydes (e.g., MG-132), which reversibly bind and block the proteasome, although they lack specificity. Peptide boronates (e.g., bortezomib) are more potent synthetic analogs of peptide aldehydes and are suitable for clinical development. Natural peptide epoxyketones (e.g., epoxomicin) are irreversible inhibitors highly specific for proteasome. β-Lactones (e.g., lactacystin) are also natural irreversible inhibitors, but they are active after hydrolysis to clasto-lactacystin β-lactone in an aqueous solution (de Bettignies and Coux 2010). Preclinical studies in cell culture and animal models documented the anti-tumor potential of proteasome inhibitors against many types of cancer cells (Zanotto-Filho et al. 2012; Mehta et al. 2015; Honma et al. 2014; Laporte et al. 2017), including melanoma (Fernández et al. 2005; Sorolla et al. 2008; Reuland et al. 2012; Selimovic et al. 2013). Two proteasome inhibitors—bortezomib and carfilzomib—were approved for multiple myeloma and mantle-cell lymphoma treatment (Adams and Kauffman 2004; Berenson et al. 2016), and other ones are undergoing clinical trials. Solid tumors, however, are much more resistant to proteasome inhibition, indicating that mechanisms through which these agents act are not fully understood.

Apart from the inhibition of transcription factor NFκB, which remains controversial (Crawford et al. 2011), the main mechanisms of proteasome inhibitor cytotoxicity in cancer cells are induction of apoptosis and disruption of the cell cycle (Crawford et al. 2011; Frankland-Searby and Bhaumik 2012; Wojcik 2013). Studies in melanoma have identified two Bcl-2 family proteins: proapoptotic Noxa and antiapoptotic Mcl-1 as the targets for bortezomib (Fernández et al. 2005; Wolter et al. 2007; Reuland et al. 2012; Selimovic et al. 2013). Proteasome inhibition also interferes with the cell cycle progression, which is regulated through the expression, ubiquitylation, and degradation of cyclins and CDK inhibitors (Starostina and Kipreos 2012). Cell cycle dysregulation and accumulation of negative regulators of cell cycle, such as CDK inhibitors p21^Cip1/Waf1^ and p27^Kip1^ following proteasome inhibition, have been previously reported in many types of cancer cells, including melanoma (Yerlikaya and Erin 2008). Finally, the inhibition of proteasome results in massive accumulation of misfolded proteins and induces synthesis of heat shock proteins (HSP). Because these molecular chaperones play a cytoprotective role and help to rescue cells from different stress-induced apoptosis, overexpressed HSPs are anti-apoptotic in function (Calderwood and Gong 2016).

Since most studies on melanoma utilizes bortezomib, which, according to the published data, has had no success in clinical trials on melanoma (Markovic et al. 2005; Obrist et al. 2015), we have decided to compare the action of structurally different proteasome inhibitors other than bortezomib, namely, MG-132, epoxomicin, and lactacystin. In this work, we analyze the effect of proteasome inhibition on a specific form of melanoma lacking melanin synthesis—the amelanotic melanoma. This form is rare but more aggressive than melanotic melanoma. Loss of melanization often occurs in initially pigmented melanomas, as they become more invasive and is associated with poor prognosis (Cheung et al. 2012). In our hamster model of melanoma (Bomirski melanomas), loss of pigment in the Ab cell line is accompanied by a faster growth rate, higher tumorigenicity, and shorter animal survival time compared to its melanotic counterpart (Ma line) (Cichorek 2011). Therefore, we examined the susceptibility of amelanotic Ab melanoma cells to experimentally triggered proteasome inhibition and analyzed the cells’ response in terms of the proliferation, apoptosis induction (with special regard to Bcl-2 family members and HSP), and cell cycle disruption.

## Materials and methods

### Animals

Male Syrian (golden) hamsters *Mesocricetus auratus Waterhouse* (3–4 months old) were used. Experimental procedures were approved by the Animal Ethics Committee at Medical University of Gdansk and conducted in accordance with National Health and Medical Research Council’s guide for the care and use of laboratory animals.

### Transplantable hamster’s melanomas

Original transplantable melanotic melanoma (Ma) has been derived from a spontaneous melanoma of the skin that appeared in a bred of golden hamster in 1959. Amelanotic melanoma line (Ab) originated from the Ma form by a spontaneous alteration (Bomirski et al. 1988). Once established, amelanotic line is maintained in vivo by consecutive, subcutaneous injection of tumor cells every 11 days. This melanoma model is known as Bomirski hamster’s melanoma.

### Isolation of amelanotic melanoma cells (Ab cells)

Ab cells were isolated from the solid tumors by a non-enzymatic method reported previously (Cichorek et al. 2007). Cell suspension contained 95–98% of viable cells as estimated by trypan blue exclusion assay. After isolation, the cells were cultured for 24 h under standard conditions (RPMI 1640, 10% fetal bovine serum (FBS), antibiotics; 37 °C at 5% CO_2_) before subsequent experiments. Each independent experiment was performed on the cells isolated from a single tumor.

### Proteasome and caspase inhibitor treatment

Ab cells were incubated with proteasome inhibitors: epoxomicin, MG-132, or lactacystin at a concentration ranging from 0.1 to 10 μM for 6 to 72 h at the standard conditions. Untreated (control) samples were treated with solvent only: DMSO for epoxomicin and MG-132 and ddH_2_0 for lactacystin. When using pan-caspase inhibitor BAF (Boc-D-FMK) (Calbiochem, USA), Ab cells were preincubated with BAF for 2 h before exposure to proteasome inhibitors.

### Cell viability assay

Cell viability was determined by XTT assay (Roche, Germany), which measures ability of the cells to reduce tetrazolium salt XTT (2,3-bis-(2-methoxy-4-nitro-5-sulfophenyl)-2*h*-tetrazolium-5-carboxanilide) to water-soluble formazan product. Ab melanoma cells were seeded in 96-well plates at a concentration 2 × 10^4^ per well and 5 h before indicated time XTT solution was added. Orange-colored formazan product was quantified at 450 nm using a microplate reader Multiscan FC (Thermoscientific, USA).

### Chymotrypsin-like proteasome activity assay

Chymotrypsin-like (ChT-L) proteasome activity was analyzed by luminescent assay (Promega, USA). According to the manufacturer’s instruction, Ab cells were seeded in 96-well plates at a concentration 10^4^ per well and incubated for 2 h. Fifteen minutes before the measurement, buffer containing luminogenic ChT-L proteasome substrate Suc-LLVY-aminoluciferin (final concentration 20 μM) and luciferase was added. A luminescent signal generated by cleavage of proteasome substrate was detected using a microplate luminometer Fluoroscan FC (ThermoScientific, USA).

### Cell cycle analysis

Cell cycle distribution was determined by flow cytometry method based on the DNA content in cells’ nuclei, as described earlier (Cichorek 2011). Ethanol-fixed 1 × 10 ^6^ Ab cells were suspended in 1 ml of the staining solution containing 40 μg/ml propidium iodide (PI, Sigma Chemicals, USA) and 100 μg/ml RNaze A and incubated 30 min at 37 °C. Fluorescence was measured using a FACS Calibur flow cytometer (Becton Dickinson Immunocytometry Systems, USA; Department of Pathophysiology, Medical University of Gdansk). A total of 20,000 events were stored from each stained sample and analyzed off-line using WinMDI2.6 software (obtained from J. Trotter, The Scripps Institute, La Jolla, USA). Cells in S and G2/M phases of the cell cycle were regarded as dividing cells and analyzed together.

### Hoechst staining

Apoptotic morphology of cell nuclei was analyzed by Hoechst 33342 (Invitrogen, USA) staining. Ab cells were incubated with 3 μg/ml Hoechst 33342 for 30 min and assessed on a reversed microscope Nikon Eclipse TE300 (Nikon, Japan) under ×10 and ×40 objective lenses.

### Phosphatidylserine externalization assay

Early apoptotic change of plasma membrane structure, phosphatidylserine (PS) externalization, was determined by Annexin V-FITC and PI staining (BD Pharmingen, USA) according to the manufacturer’s instructions. Cells were seeded in 12-well plates at a concentration 1 × 10^6^ per well. Staining allows to determine three populations of cells: viable (An−/PI−), early apoptotic (An+/PI−), and late apoptotic (An+/PI+). Fluorescence intensity was measured by a FACS Calibur flow cytometer (Becton Dickinson Immunocytometry Systems, USA; Department of Pathophysiology, Medical University of Gdansk). A total of 20,000 events were stored from each stained sample and analyzed off-line using WinMDI2.6 software.

### Immunoblotting

Total cell lysates were obtained by incubation of 3 × 10^6^ cells in lysis buffer (10 mM Tris-HCl pH 8.0, 140 mM NaCl, 2% TX-100) with addition of the protease inhibitors (1 mM AEBSF, 0.8 μM aprotinin, 50 μM bestatin, 15 μM E-64, 20 μM leupeptin, 10 μM pepstatin A) for 1 h on ice and cenrifugation for 15 min at 14,000 rpm. Supernatants were collected and stored at −70 °C until further processing. Total protein amount was quantified by Lowry assay (Bio-Rad, USA). Sixty micrograms of lysate was subjected to electrophoresis in 10%, 12% or gradient 4–15% SDS gels under reducing conditions and transferred to nitrocellulose membrane (Bio-Rad, USA). After 2-h blocking in 4% non-fat milk, membranes were probed overnight with primary antibodies: rabbit polyclonal anti-caspase 3 (Santa Cruz Biotechnology, USA; 1:250), mouse monoclonal anti-caspase 9 (Stressgen, USA; 1:500), rabbit polyclonal anti-HSP27 (Stressgen, USA; 1:1000), mouse monoclonal anti-HSP70 (StressMarq, USA; 1:5000), mouse monoclonal anti-p21 (BD Pharmingen, USA; 1:250), mouse monoclonal anti-p27 (BD Pharmingen, USA; 1:1000), mouse monoclonal anti-Noxa (Abcam, UK; 1:200), and rabbit monoclonal anti-Mcl 1 (Abcam, UK; 1:500). Anti-β-actin mouse monoclonal antibody (Sigma Chemicals, USA; 1:15,000) was used as an equal protein loading control. After washing, membranes were next incubated for 2 h with horseradish peroxidase-conjugated secondary antibody (Pierce, USA; 1:50,000). Chemiluminescent signal was developed using the SuperSignal West Pico system (Pierce, USA) and visualized on X-ray film. Band intensity was semiquantified using ImageJ 1.38 software (National Institutes of Health, USA) and was shown as a ratio to actin.

### Cytochrome C and AIF immunofluorescent staining

For immunofluorescent detection of cytochrome C and apoptosis-inducing factor (AIF), cells were cultured on coverslips with proteasome inhibitors for 6 h. Double staining of mitochondria and cytochrome C was performed to analyze cytochrome C release from mitochondria to cytoplasm. First, the mitochondria in living cells were stained with 250 nM MitoTracker Red CMXRos (Invitrogen, USA) for 30 min. Next, the cells were 10 min fixed (3.7% formaldehyde), 5 min permeabilized (90% methanol), PBS rinsed, 30 min blocked in 3% FBS, and probed for 1 h with mouse monoclonal anti-cytochrome C antibody (clone 6H2.B4, BD Pharmingen, USA; 1:400). After PBS rinsing, the cells were incubated with AlexaFluor 488-conjugated goat anti-mouse secondary antibody (Invitrogen, USA; 1:400) for 1 h.

To determine AIF translocation, double staining of cell nuclei and AIF was performed. The cells were 10 min fixed (4% paraformaldehyde), 10 min permeabilized (0.2% Triton), PBS rinsed, blocked in 2% bovine serum albumin (BSA) for 30 min. Next, the cells were incubated overnight in 4 °C with rabbit monoclonal anti-AIF antibody (clone E20, Abcam, UK; 1:500). After PBS rinsing, the cells were incubated with AlexaFluor 488-conjugated goat anti-rabbit secondary antibodies (Invitrogen, USA; 1:400) for 1 h. To remove RNA before staining of cell nuclei by PI, the cells were treated by 0.1 mg/ml RNase A (Sigma Chemical, USA) in 37 °C for 30 min, PBS rinsed, and then stained in 2.5 μg/ml PI solution in PBS for 30 min. Finally, the stained cells on coverslips were mounted in PermaFluor (Shanon Lipshaw, USA) as the microscope slides. Immunofluorescent staining was assessed using a confocal laser scanning microscopy system (Radiance 2100, Bio-Rad UK) mounted on a microscope Eclipse 600 (Nikon, Japan). Images were obtained with ×60 oil immersion objective lenses and analyzed with the LaserSharp 2000 and LaserPix v. 2.0 software (both Bio-Rad, UK).

### Statistical analysis

The statistical analysis was performed using the data analysis software system STATISTICA version 12, StatSoft Inc. (2014). Nonparametric Mann-Whitney *U* test, Kruskall-Wallis, and Dunn tests were used. *p* ≤ 0.05 was considered statistically significant. Data are displayed as arithmetical mean ± standard deviation (SD).

## Results

### Proteasome inhibition decreases viability of Ab melanoma cells

**Fig. 1:**
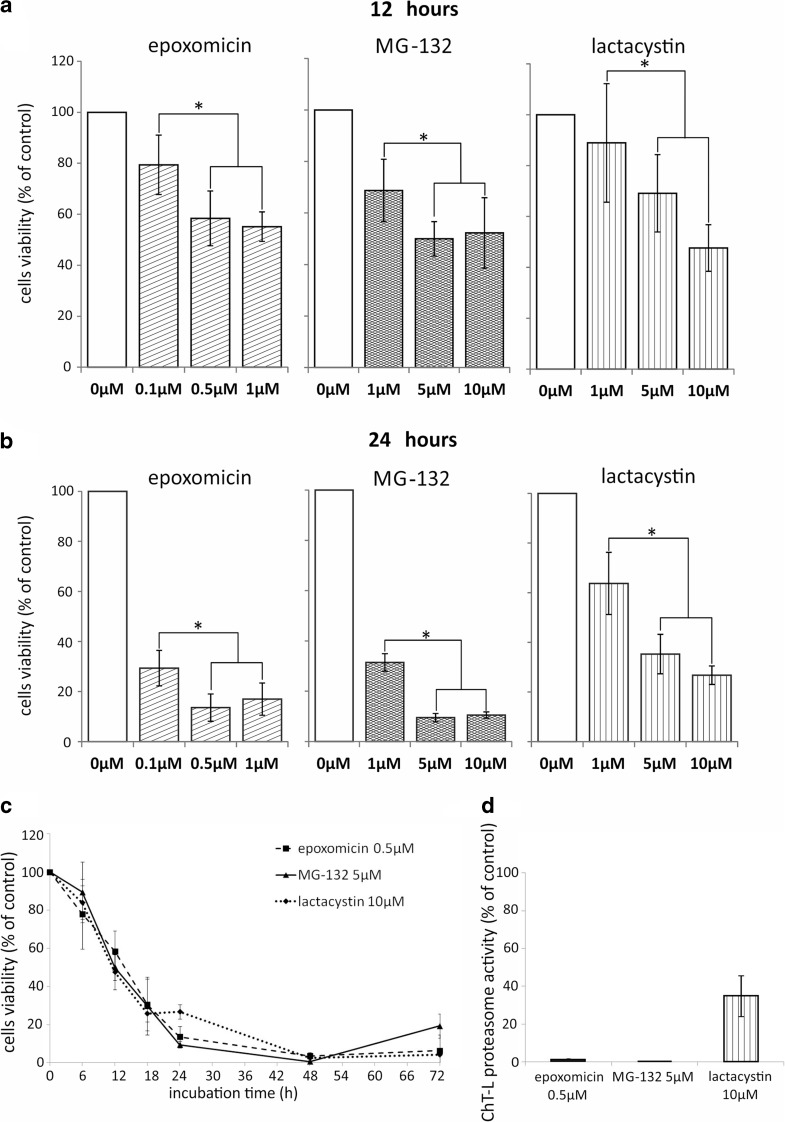
Cytotoxic effect of proteasome inhibitors on Ab melanoma cells. Cell viability was determined by XTT assay and expressed as a percentage of the viability of untreated cells. Cells were exposed to increasing concentrations of epoxomicin, MG-132, or lactacystin for 12 (**a**) and 24 (**b**) hours or to 0.5 μM epoxomicin, 5 μM MG-132, or 10 μM lactacystin for up to 72 h (**c**). An *asterisk* (*) indicates a statistically significant difference between various proteasome inhibitor concentrations; *p* < 0.05, Kruskal-Wallis test. **d** Inhibition of chymotrypsin-like (ChT-L) proteasome activity in Ab melanoma cells. Cells were exposed to 0.5 μM epoxomicin, 5 μM MG-132, or 10 μM lactacystin for 2 h, and proteasome activity was measured using a luminescent assay. The data represent means ± SD of three independent experiments

Ab cells were exposed to increasing concentrations of three structurally different proteasome inhibitors: epoxomicin, MG-132, and lactacystin for up to 72 h and cell viability changes were analyzed by XTT reduction. All the compounds tested diminished Ab cell viability in a dose-dependent manner (Fig. 1a, b). During the initial 6 h of treatment, only minor changes were observed, since neither of the inhibitors reduced the cell viability more than 20% of the control value (data not shown). After 12 h, the lowest concentrations of inhibitors (0.1 μM epoxomicin, 1 μM MG-132, and 1 μM lactacystin) decreased Ab cell viability to 79, 69, and 89% of the control, respectively (Fig. 1a). The five times’ higher doses reduced the cell viability a further 20% (Fig. 1a; *p* < 0.05). Twenty-four-hour treatment with the lowest concentration of the inhibitors decreased cell viability to 29, 31, and 63%, respectively (Fig. 1b). Again, five times higher doses were significantly more cytotoxic and left only 13, 9, and 35% of viable cells (Fig. 1b). A further increase in the inhibitor concentration did not change their cytotoxicity significantly (Fig. 1a, b). At all time points, epoxomicin was the most potent of the three proteasome inhibitors tested. At a concentration of 0.5 μM, its effect was comparable with the action of a ten times higher dose of MG-132 (5 μM) or a 20 times higher dose of lactacystin (10 μM) (Fig. 1c). The least effective of the proteasome inhibitors was lactacystin. Twenty-four-hour incubation with its 10 μM concentration left 27% cells alive, and similar viability level was observed at that time for 1 μM MG-132 or 0.1 μM epoxomicin (Fig. 1b). Finally, 48-h exposure, regardless of the proteasome inhibitor used, resulted in the death of over 95% of the cells (Fig. 1c).

It is worth noting that 2-h exposure to 0.5 μM epoxomicin and 5 μM MG-132 totally blocked chymotrypsin-like (ChT-L) proteasome activity in Ab cells (Fig. 1d), while lactacystin, even in dose of 10 μM, did not inhibit this activity completely, but decreased it to 35% of the control value. Higher concentrations of lactacystin were not tested because of the possible unspecific inhibition of the other cellular proteases.

Because epoxomicin exerted the strongest cytotoxic effect on Ab melanoma cells, it was chosen for further investigations concerning the molecular mechanisms of cell death induced by proteasome inhibition. Epoxomicin was used in 0.5 μM concentration, which was the lowest dose that totally blocked ChT-L proteasome activity (Fig. 1d).

### Epoxomicin induces apoptosis in Ab melanoma cells

**Fig. 2:**
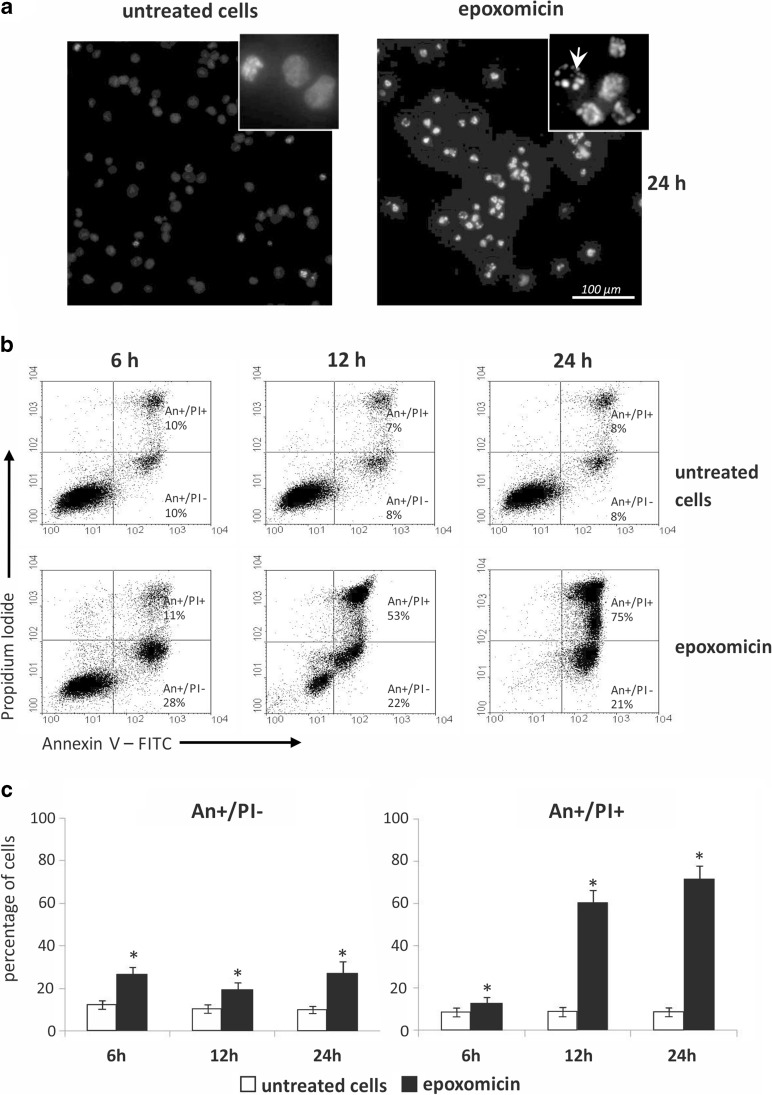
Proteasome inhibition-induced apoptotic cell death changes in Ab melanoma cells. Cells were exposed to 0.5 μM epoxomicin for 6, 12, and 24 h. **a** Hoechst 33342 staining of Ab cells after 24 h of treatment. Fluorescence of apoptotic nuclei is more intense than in viable cells. Chromatin condensation and nucleus fragmentation are prominent after treatment (*arrow*). **b** Apoptotic plasma membrane changes in Ab cells. Cells were stained with FITC-conjugated annexin V (An) and propidium iodide (PI) to estimate early (An+/PI−) and late apoptotic (An+/PI+) cells. Representative dot plots from flow cytometry analysis results are shown. **c** Increase in early (An+/PI−) and late (An+/PI+) apoptotic Ab melanoma cells after exposure to epoxomicin. The data represent means ± SD of three independent experiments. An *asterisk* (*) indicates a statistically significant increase in apoptotic cells in comparison to untreated cells, *p* < 0.05, Kruskal-Wallis test

Hoechst 33342 staining revealed a typical apoptotic morphology of cell nuclei exposed to epoxomicin (such as chromatin condensation and nucleus fragmentation) (Fig. 2a). Since about 10% of Ab cells undergoes spontaneous apoptosis, single apoptotic nuclei were also observed in untreated cells, but after 24 h of epoxomicin treatment, the number of cells with apoptotic nuclei markedly increased (Fig. 2a). Epoxomicin also induced phosphatidylserine externalization (An + cells), which is the marker of apoptotic changes in the plasma membrane (Fig. 2b, c). In untreated cells, the spontaneous early (An+/PI−) and late (An+/PI+) apoptotic cells comprised about 10% each. A statistically significant response to epoxomicin was detected as early as after 6 h when the number of early apoptotic cells (An+/PI−) rose to over 20% (Fig. 2c, *p* < 0.05) and remained at this level until 24 h. The number of late apoptotic cells (An+/PI+) under epoxomicin treatment increased significantly to 60% after 12 h and 70% after 24 h (Fig. 2c).

**Fig. 3:**
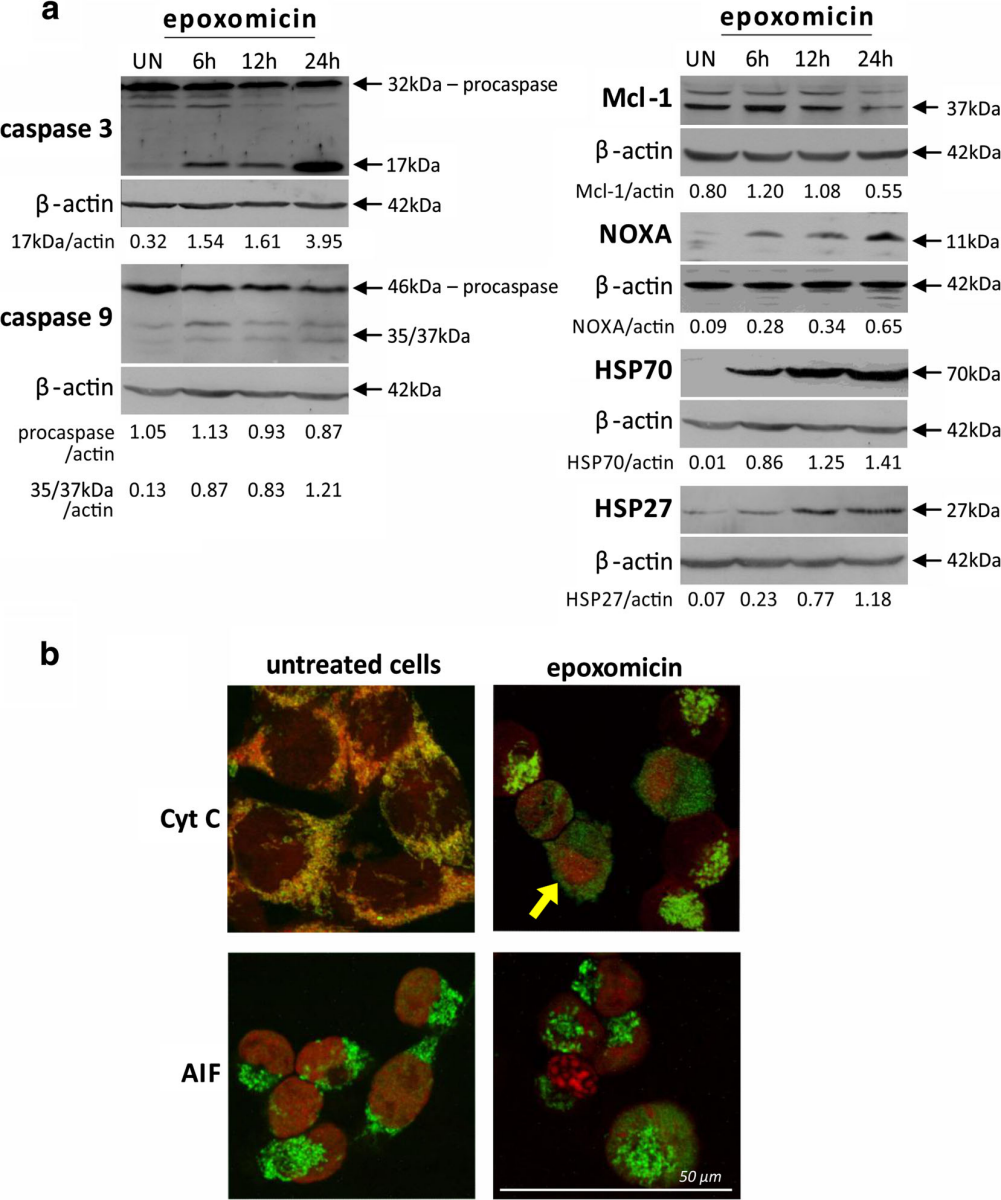
Epoxomicin-induced mitochondrial pathway of apoptosis and HSP expression in Ab melanoma cells. Cells were exposed to 0.5 μM epoxomicin for 6, 12, and 24 h. **a** Immunoblot analysis of caspase 3 and 9 activation and expression of Mcl-1, Noxa, and HSP proteins in Ab cells in response to epoxomicin. β-Actin was used as a control of the equal protein loading. *UN* untreated cells. The densitometric ratio of band intensity is shown. **b** Double-labeled immunofluorescent staining revealing cytochrome C and AIF release from mitochondria in Ab cells treated with epoxomicin for 6 h. *Upper panel* cytochrome C (*green*) and mitochondria (*red*), an *arrow* indicates the membrane blebbing specific for apoptotic cell death; *lower panel*: AIF (*green*) and cell nucleus (*red*)

The apoptotic death of Ab cells exposed to epoxomicin was further confirmed by caspase 3 activation. Procaspase 3 activation (detected by the presence of 17 kDa cleaved subunit) started at 6 h and lasted throughout the duration of the experiment, as shown by immunoblot studies (Fig. 3a). Classic apoptotic cell morphology was also observed in cytochrome C immunofluorescent staining, where membrane blebbing was evident (Fig. 3b, arrow indication).

### Epoxomicin activates the mitochondrial apoptotic pathway in Ab melanoma cells

Immunoblot analysis of initiator caspase 9 revealed its activation, estimated by the presence of the active subunits 35/37 kDa, after 6 h of exposure to epoxomicin. The procaspase 9 (46 kDa) protein level gradually declined with time up to 24 h (Fig. 3a). Induction of the mitochondrial pathway of apoptosis was confirmed by immunofluorescent detection of cytochrome C and AIF translocation from mitochondria (Fig. 3b). In untreated cells, cytochrome C and AIF were localized in mitochondria, but after 6 h of epoxomicin exposure, both proteins were released to cytosol (Fig. 3b).

Ab cells express antiapoptotic factors such as survivin or Bcl-2 (unpublished data). In this work, we examined two other proteins of Bcl-2 family: proapoptotic Noxa and antiapoptotic Mcl-1. Immunoblot analysis presented in Fig. 3a showed that exposure to epoxomicin induced Noxa protein expression and its accumulation with time. Mcl-1, as the prosurvival protein, was constitutively expressed in untreated Ab cells, and proteasome inhibition caused its transient increase at 6 h of treatment (Fig. 3a).

Furthermore, proteasome inhibition resulted in the accumulation of anti-apoptotic proteins from the HSP family: HSP70 and HSP27. We found no constitutive expression of HSP70 and a very low level of HSP27 in untreated Ab cells (Fig. 3a). Proteasome inhibition led to rapid and massive HSP70 up-regulation, which started 6 h after epoxomicin treatment. Induction of HSP27 expression was weaker and became apparent after 12 h.

### Epoxomicin induces apoptosis in both caspase-dependent and caspase-independent way

**Fig. 4:**
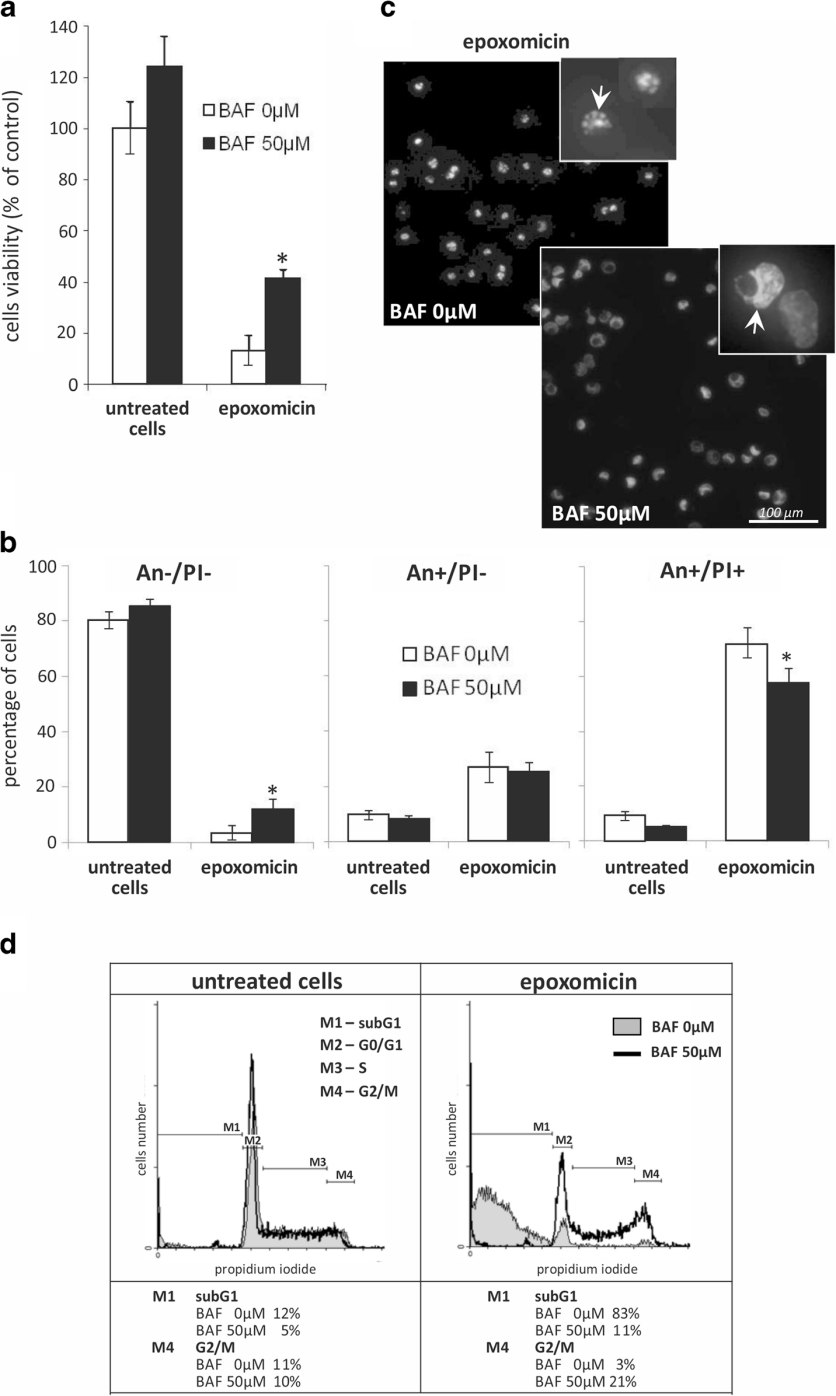
Epoxomicin-induced apoptosis of Ab melanoma cells only partially relies on caspase activation. Ab cells were treated with 0.5 μM epoxomicin alone or in the presence of 50 μM pan-caspase inhibitor BAF for 24 h. **a** Cell viability was measured by an XTT assay and calculated as a percentage of the viability of untreated cells. **b** Cells were stained with FITC-conjugated annexin V (An) and propidium iodide (PI) to estimate live cells (An−/PI−), early (An+/PI−) and late apoptotic (An+/PI+) cells. In **a** and **b**, the data represent the means ± SD of three independent experiments; *asterisk* (*) indicates statistically significant difference between BAF 0 μM and BAF 50 μM, Mann-Whitney *U* test, *p* < 0.01 (**a**) or *p* < 0.05 (**b**). **c** Ab cells were treated with epoxomicin alone or in the presence of BAF and stained with Hoechst 33342. *Arrows* indicate the nucleus fragmentation in the absence of BAF (*upper panel*, BAF 0 μM) and chromatin condensation, but no nucleus fragmentation in the presence of BAF (*lower panel*, BAF 50 μM). **d** Cell cycle distribution was determined in PI-stained Ab cells by flow cytometry. Representative histograms are shown. The percentage of cells with fragmented DNA (subG1, M1) and in the G2/M (M4) phase of the cell cycle are indicated below the corresponding histograms

**Fig. 5:**
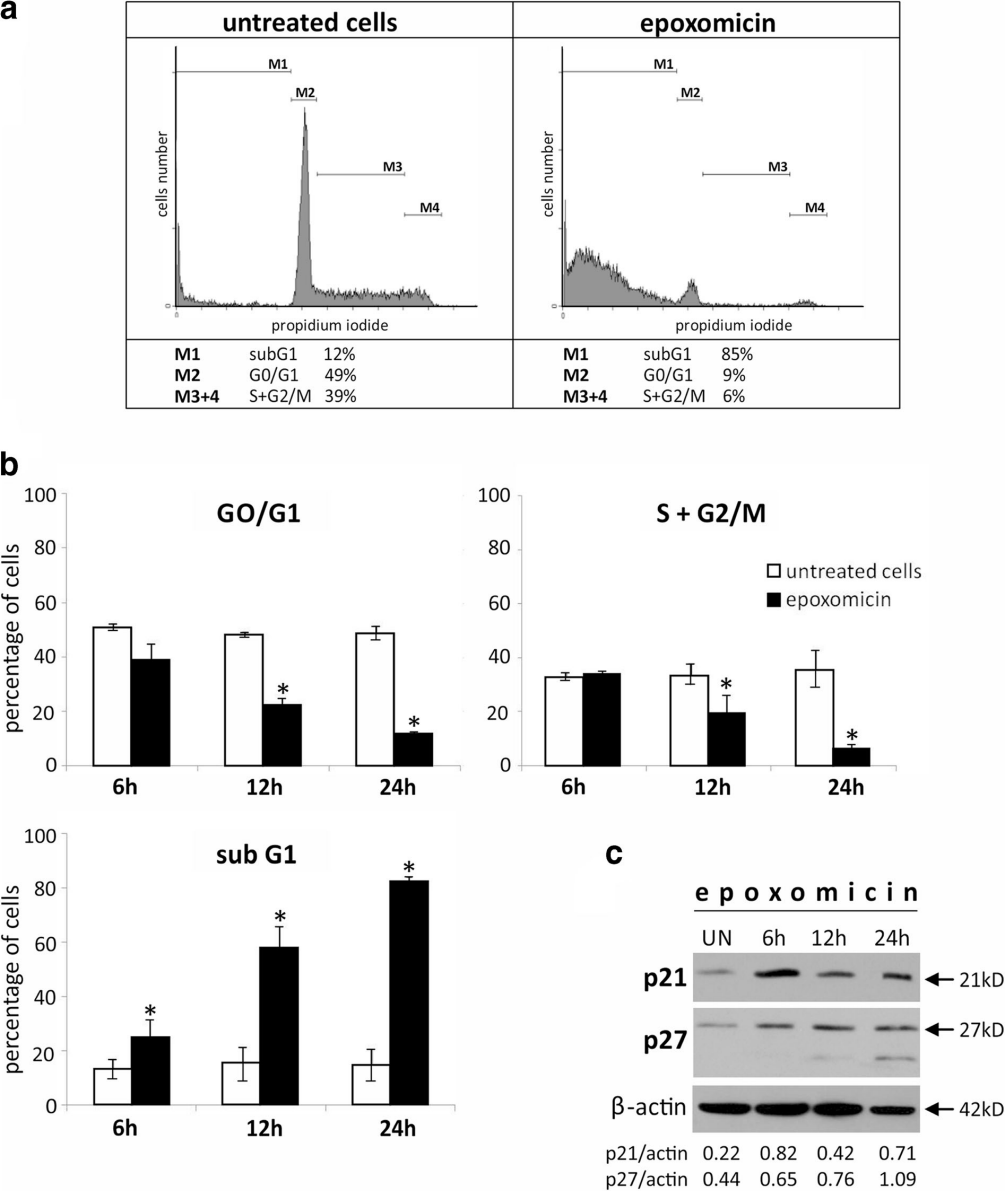
Ab melanoma cell cycle analysis and changes in the cdk (cell cycle-dependent kinases) inhibitors p21^Cip1/Waf1^ and p27^Kip1^ after epoxomicin treatment. Cells were exposed to 0.5 μM epoxomicin for up to 24 h. **a** Representative histograms of cell cycle analysis of cells exposed to epoxomicin for 24 h. DNA content was estimated by propidium iodide staining, and cell cycle distribution was assessed by flow cytometry. **b** Time-dependent decrease in G0/G1 and S + G2/M phases and accumulation of destroyed cells in subG1. Data represent means ± SD of three independent experiments. *Asterisks* (*) indicate statistically significant difference between untreated and epoxomicin-treated cells (*p* < 0.05), Kruskal-Wallis test. **c** Immunoblot analysis of p21^Cip1/Waf1^ and p27^Kip1^ up-regulation in Ab cells treated with 0.5 μM epoxomicin. β-actin was used as a control of the equal protein loading. UN-untreated cells. The densitometric ratio of band intensity is shown

To verify whether apoptotic Ab melanoma cell death following proteasome inhibition relies entirely on caspase activity, the cells were preincubated with 50 μM pan-caspase inhibitor Boc-D-FMK (BAF) and then exposed to 0.5 μM epoxomicin. Caspase inhibition resulted in the significant protection of the cells against epoxomicin cytotoxicity as revealed by the increase in viable cells from 13% after 24 h of expoxomicin treatment to about 40% (*p* < 0.01), following the same conditions in the presence of BAF (Fig. 4a). BAF did not affect the early apoptotic changes in the plasma membrane, since there was no difference in the number of An+/PI− cells after 24-h exposure to epoxomicin alone or in the presence of BAF (Fig. 4b). However, inhibition of caspases seemed to slow down the progression of apoptosis because fewer cells entered the late stage of apoptotic cell death (An+/PI+) and significantly more remained viable (An−/PI−) (Fig. 4b, *p* < 0.05). Additionally, Hoechst 33342 staining showed that caspase inactivation by BAF inhibits fragmentation of apoptotic cell nuclei (Fig. 4c). These changes were further confirmed by flow cytometric analysis of DNA content and cell cycle distribution (Fig. 4d). BAF prevented DNA fragmentation and therefore reduced accumulation of subG1 population after 24 h of epoxomicin treatment. In the presence of BAF, the percentage of cells with decreased DNA content (subG1 cells) significantly declined from over 80 to 11% (Fig. 4d, *p* < 0.01). As shown in Fig. 5, proteasome inhibition alone caused no cell cycle arrest but did cause subG1 accumulation, whereas caspase inhibition by BAF during epoxomicin exposure resulted in the accumulation of cells in the G2/M phase. Cell cycle distribution analysis revealed 20% of Ab cells in the G2/M phase following 24-h exposure to epoxomicin and BAF in comparison to only 3% after treatment with epoxomicin alone (Fig. 4d, M4).

### Epoxomicin does not arrest Ab melanoma cell cycle

Flow cytometric analysis of propidium iodide-stained cells revealed that proteasome inhibition by epoxomicin did not accumulate Ab melanoma cells in the S + G2/M phases (Fig. 5a; M3, M4). On the contrary, even though at 6 h the number of cells in the S + G2/M phases did not change markedly, remaining over 30%, later it began to decrease and finally declined to 6% after 24 h (Fig. 5b; *p* < 0.05). With time, epoxomicin also markedly reduced the G0/G1 cell content (Fig. 5a, b). Reduction of cells in G0/G1 and S + G2/M phases was accompanied by the accumulation of the subG1 population, which comprises the cells with reduced DNA content: cells with fragmented nuclei and apoptotic bodies (Fig. 5a, M1). Epoxomicin treatment significantly increased subG1 population from about 10% in untreated cells to about 80% after 24-h exposure (Fig. 5a, b). Accumulation of subG1 population corresponds with decreased cell viability measured by XTT reduction (Fig. 1b) and apoptotic changes in cell nuclei revealed by Hoechst 33342 staining (Fig. 2a).

Immunoblot analysis of the inhibitors of cycle-dependent kinases, p21^Cip1/Waf1^ and p27^Kip1^, revealed the transient accumulation of both proteins (Fig. 5c).

## Discussion

In this work, we examine the cytotoxic effect of three structurally different proteasome inhibitors: MG-132, epoxomicin, and lactacystin on amelanotic melanoma of hamster origin (Ab cells). We also explore molecular mechanisms of apoptosis induction and cell cycle disruption elicited by epoxomicin, which we found to be the most potent proteasome inhibitor against amelanotic melanoma.

### Sensitivity of Ab melanoma cells is different for various proteasome inhibitors

Among the proteasome inhibitors examined, the most effective was epoxomicin, while the action of lactacystin was the weakest. Similar differences in cell sensitivity were shown previously in human melanoma cell lines, which were more sensitive to epoxomicin than to MG-132 or to MG-132 than lactacystin (Sorolla et al. 2008; Qin et al. 2005). It seems that the cytotoxic potential of epoxomicin is related to its high specificity and irreversible binding to proteasome (de Bettignies and Coux 2010). The relative resistance of Ab cells to lactacystin probably results from the incomplete inhibition of proteasome activity, which in our study was not achieved even for 10 μM drug concentration. We may only hypothesize that this is due to the specific chemical structure of β-lactone. Lactacystin is not active itself, but in an aqueous solutions, it undergoes spontaneous hydrolysis to clasto-lactacystin β-lactone, which is an active but less stable compound (de Bettignies and Coux 2010). Furthermore, hydrolysis requires neutral pH, while amelanotic melanoma cells are known for their ability to acidify the medium (Halaban 2002).

### Epoxomicin induces apoptosis in Ab melanoma cells

Because of its cytotoxic potency against Ab melanoma cells, we have chosen epoxomicin for the further investigation of the mechanisms underlying the cell’s response. Many studies have proved that the principal way of cancer cell death elicited by proteasome inhibitors is apoptosis. However, most studies on melanoma relied on bortezomib (Fernández et al. 2005; Qin et al. 2005; Wolter et al. 2007; Yerlikaya and Erin 2008; Amschler et al. 2010; Reuland et al. 2012; Selimovic et al. 2013), and little is known about the mechanisms of cell death induced by other proteasome inhibitors, such as epoxomicin. Only Sorolla et al. (2008) reported caspase activation after epoxomicin treatment in primary human melanoma cell lines. We found that in Ab cells, epoxomicin decreased viability mainly through apoptosis induction, as indicated by typical apoptotic hallmarks, e.g., chromatin condensation, externalization of phosphatidylserine, or caspase-3 activation. The caspase-9 activation as well as cytochrome C and AIF release from mitochondria showed the induction of the mitochondrial pathway of apoptosis.

AIF is also known for its role in the specific form of cell death, referred to as “necroptosis” (regulated necrosis), which does not rely on caspase activity (Galluzzi et al. 2014). AIF translocation from mitochondria indicated that the death of Ab melanoma cells might be partially caspase-independent. Indeed, inactivation of caspases did not prevent the cytotoxic effect of epoxomicin on Ab cells, but reduced it by half. This is in agreement with other studies, whose authors observed partial protection of melanoma cells by caspase inhibitors against cytotoxic effect of bortezomib (Qin et al. 2005; Sorolla et al. 2008; Selimovic et al. 2013). In our study, inhibition of caspases had little impact on the early stages of cell death, such as changes in plasma membrane, but it completely blocked the late events of cell demise, e.g., nucleus fragmentation or accumulation of cells with fragmented DNA (subG1 fraction). We also observed cell cycle arrest in G2/M phase, which was not present following epoxomicin treatment alone.

### Epoxomicin changes the expression of Bcl-2 and HSP proteins

It has been postulated that the key elements of bortezomib-induced apoptosis in melanoma cells are Bcl-2 family members—proapoptotic Noxa and antiapoptotic Mcl-1. Bortezomib has been found to trigger substantial accumulation of Noxa after 18 or 24 h in numerous melanoma cell lines (Fernández et al. 2005; Qin et al. 2005; Wolter et al. 2007; Reuland et al. 2012; Selimovic et al. 2013). We observed a similar effect in our model, where Noxa accumulation started after 6 h of epoxomicin treatment and continued throughout the time of the experiment (24 h). Stabilization of Noxa probably facilitated cytochrome C and AIF release from mitochondria in Ab cells. Accumulation of Noxa was also reported after MG-132 treatment in A375 amelanotic melanoma cells (Miller et al. 2009).

Induction of Noxa expression may be a way for proteasome inhibitors to overcome relatively high levels of the antiapoptotic proteins present in melanoma. Ab melanoma cells constitutively express survivin, Bcl-2, and Bcl-XL proteins (unpublished data). In this study, we also found the constitutive expression of Mcl-1, the prosurvival member of Bcl-2 family preferentially bounded by Noxa, and its transient accumulation after epoxomicin treatment. Our results are in accordance with studies on the bortezomib effect in different melanoma cell lines, which revealed a rapid increase in Mcl-1 expression at first and then its reduction to or below basal levels (Wolter et al. 2007). Mcl-1 accumulation was also found in melanoma cell line A375 after 24-h treatment with MG-132 (Miller et al. 2009).

Other antiapoptotic proteins commonly overexpressed in many types of cancers including melanoma are HSP70 and HSP27 (Shipp et al. 2013; Calderwood and Gong 2016). As molecular chaperones, HSP70 and HSP27 are strongly up-regulated in response to proteasome inhibition in various types of cancer cells (Kim et al. 2011; Shah et al. 2016). In Ab cells, we found that protein accumulation after epoxomicin treatment was particularly massive in the case of HSP70 and this protein seemed not to be constitutively expressed in these cells. HSP27 was present in untreated Ab cells at a very low level and its accumulation was slower. HSP70 and HSP27 status is different in various melanoma models. BML, A375, SK-Mel-19, and SK-Mel-103 cells showed no constitutive expression of HSP70, but it very efficient induction by bortezomib (Fernández et al. 2005; Selimovic et al. 2013). In another study, HSP70 was found to be constitutively expressed in B16F10 cells and only weakly up-regulated by bortezomib (Yerlikaya and Erin 2008). There is no data on HSP27 changes in melanoma following proteasome inhibition, but studies on the other cancer cell lines revealed either no changes, as in HL-60 and K562 cells (Klikova et al. 2015) or its accumulation in response to bortezomib or MG-132, in the case of PC-3 cells (Kumano et al. 2012). In our model, the overexpression of both HSP70 and HSP27 was apparently not sufficient to rescue Ab melanoma cells from epoxomicin-induced apoptosis.

### Epoxomicin does not arrest the Ab melanoma cell cycle

Proteasome inhibition can disrupt the cell cycle and consequently induce apoptosis also by targeting the cell cycle control factors (Crawford et al. 2011; Frankland-Searby and Bhaumik 2012). Various proteasome inhibitors, such as MG-132, bortezomib, and carfilzomib, have been proven to stop the cell cycle of different tumor cells (Zanotto-Filho et al. 2012; Mehta et al. 2015), including melanoma (Fernández et al. 2005; Amschler et al. 2010), in a phases S and G2/M. In our study, however, there was no accumulation of Ab cells in the S + G2/M phase after epoxomicin treatment. Instead, even as early as 6 h after exposure, we noticed a significant accumulation of the subG1 population, which comprises cells with a reduced DNA content. The cell cycle was blocked only after inactivation of caspases which blocked late stages of apoptosis (nucleus fragmentation and apoptotic body formation). Consequently, caspase inhibition during epoxomicin treatment resulted in a subG1 fraction decrease and cell cycle arrest in the G2/M phase. The same effect of caspase inactivation was reported by Sorolla et al. (2008) in bortezomib-treated melanoma cells.

To better understand the lack of cell cycle arrest following exposure to epoxomicin alone, we estimated the level of key cdk inhibitors p21^Cip1/Waf1^ and p27^Kip1^ in Ab cells. The up-regulation of these proteins has been shown to accompany cell cycle arrest induced by bortezomib or MG-132 in different types of cancer cells (Zanotto-Filho et al. 2012; Lu et al. 2008; Yang et al. 2012) including melanoma (Yerlikaya and Erin 2008). We found p21^Cip1/Waf1^ and p27^Kip1^ accumulation in Ab cells after epoxomicin treatment, even though their cell cycle was not arrested. The cytotoxic action of epoxomicin is probably potent enough to overcome stabilization of p21^Cip1/Waf1^ and p27^Kip1^ proteins and induce apoptosis without cell cycle disruption.

**Fig. 6:**
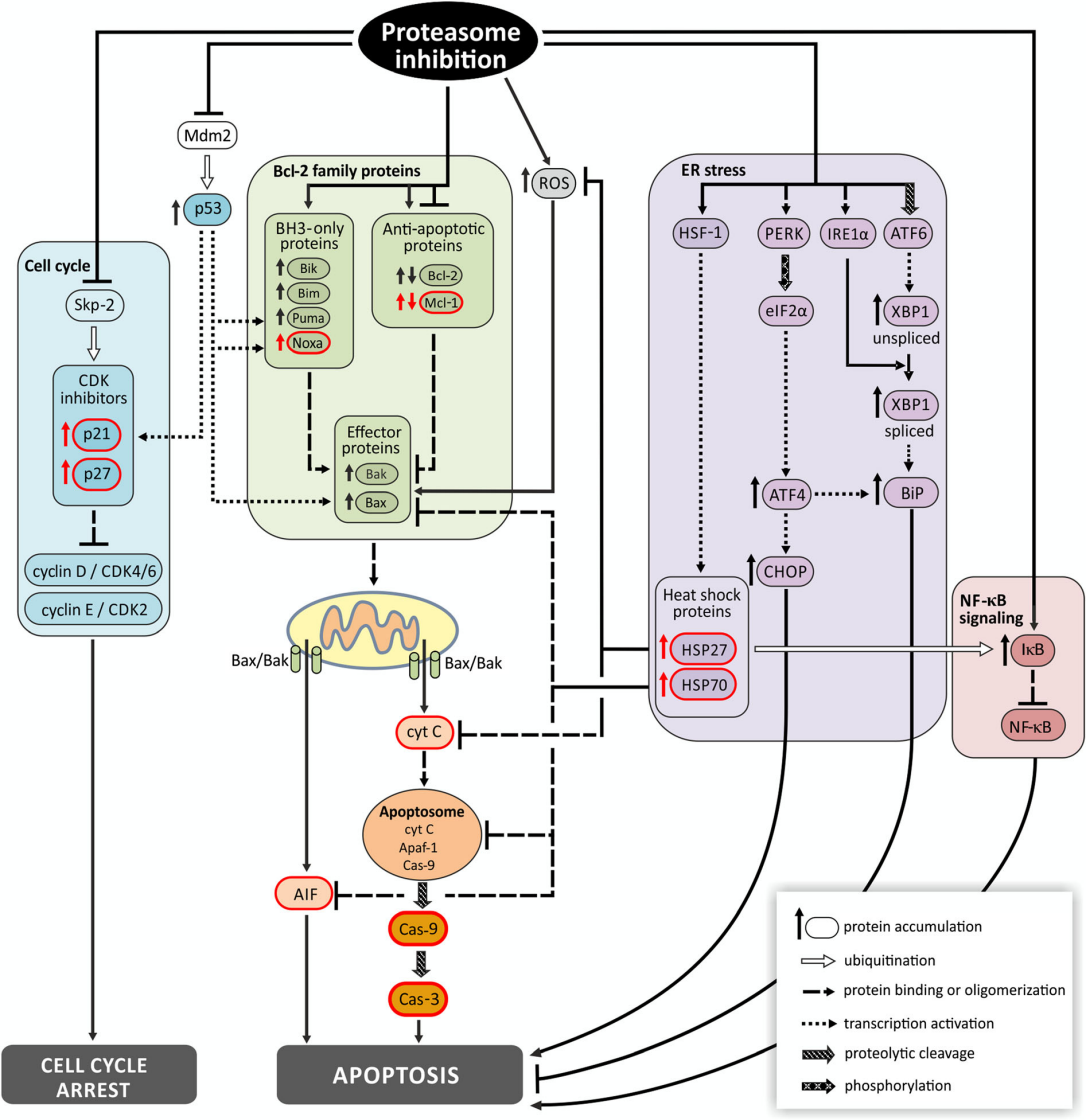
Principal mechanisms of action of proteasome inhibitors in cancer cells including melanoma. Principal molecular mechanisms underlying cytotoxic effect of proteasome inhibition involve the following: (i) up-regulation of pro-apoptotic Bcl-2 family proteins, (ii) stabilization of CDK inhibitors, (iii) p53 stabilization, (iv) endoplasmic reticulum (ER) stress, (v) decreased NF-κB signaling, and (vi) oxidative stress (accumulation of ROS). These changes result in cell cycle arrest and apoptosis in cancer cells. Inhibition of proteasome activity increases expression of pro-apoptotic BH3-only members of Bcl-2 family, including Noxa, and in some models down-regulates anti-apoptotic factors such as Bcl-2 and Mcl-1. Enhanced pro-apoptotic signaling promotes the assembly of Bax-Bak oligomers in mitochondrial outer membrane, cytochrome C and AIF release to cytosol, apoptosome formation and finally activation of caspases. Apoptotic signals are augmented by p53, which is activated by ROS-induced DNA damage and further stabilized by blocking its proteasomal degradation after ubiquitination by Mdm2. Accumulated p53 activates expression of its target genes: Puma, Noxa, and Bax. p53 also arrests cell cycle by inducing expression of CDK inhibitor p21. CDK inhibitors p21 and p27 are also directly up-regulated by proteasome inhibitors, which prevent their proteasomal degradation after ubiquitination by Skp-2. Another mechanism promoting apoptosis is down-regulation of NF-κB signaling. Inhibition of proteasome prevents degradation of inhibitory protein-IκB and subsequent activation and translocation of NF-κB to the nucleus. Aggregation of misfolded proteins resulting from proteasome inhibition triggers ER stress and defense mechanism called unfolded protein response (UPR). Aggregated proteins activate ER transmembrane proteins PERK, IRE1α, and transcription factor ATF6 by their homodimerization and proteolytical processing. ER stress also triggers HSF-1 trimerization and binding to HSP promoters. This mechanism initially protects the cell by increased expression of protein chaperones HSP70, HSP27, and BiP/Grp78. Overexpressed HSP70 and HSP27 are inhibitors of apoptosis. HSP70 inhibits assembly of Bax oligomers, prevents recruitment of procaspase-9 to apoptosome, and blocks nuclear translocation of AIF and chromatin condensation. HSP27 associates with cyt C and inhibits the formation of apoptosome, decreases ROS content, and targets IκB for ubiquitination. When overwhelmed, UPR triggers apoptosis by activation of CHOP. Elements examined in the present study are shown in red. → activation, ⊣ inhibition. *AIF* apoptosis-inducing factor, *Apaf-1* apoptotic protease activating factor 1, *ATF* activating transcription factor, *Bak* Bcl-2 antagonist/killer-1, *Bax* Bcl-2-associated X protein, *Bcl-2* B cell lymphoma 2, *Bik* BCL2 interacting killer, *Bim* Bcl-2-like protein 11, *BiP/Grp78* binding immunoglobulin protein/78 kDa glucose-regulated protein, *CDK* cyclin-dependent kinase, *CHOP* CCAAT/enhancer-binding protein homologous protein, *eIF2a* eukaryotic initiation factor 2, *IκB* inhibitory protein κB, *HSF-1* heat-shock transcription factor 1, *IRE1α* inositol-requiring enzyme 1, *Mcl-1* myeloid cell leukemia 1, *NF-κB* nuclear factor κB, *PERK* ER-resident protein kinase, *Puma* p53-up-regulated modulator of apoptosis, *ROS* reactive oxygen species, *Skp-2* S-phase kinase-associated protein 2, *XBP1* X-box binding protein 1

In summary, our study has revealed the differential sensitivity of amelanotic Ab melanoma cells to three proteasome inhibitors of different chemical structures, among which epoxomicin—a member of peptide epoxyketone natural product family from an Actinomycetes strain Q996-17—was especially effective. The antitumor activity of epoxomicin was first discovered in studies against amelanotic melanoma (B16F10 cell line) (Hanada et al. 1992); the authors, however, only reported its cytotoxic effect on cell viability without any analysis of its action. In this study, we explored the cell death mechanisms elicited by epoxomicin in melanoma for the first time. We have demonstrated that Ab cells die on the mitochondrial pathway of apoptosis but also partially by a caspase-independent way of death. Apoptotic signaling is so potent that cell death is not preceded by cell cycle arrest despite p21^Cip1/Waf1^ and p27^Kip1^ stabilization. Finally, even HSP accumulation does not protect the Ab melanoma cells against epoxomicin cytotoxicity. Thus, this special sensitivity of amelanotic melanoma to epoxomicin needs further investigation. In Fig. 6, we summarize mechanisms of action of proteasome inhibitors in cancer cells, including melanoma, with elements examined in the present study shown in red.
